# Climber’s muscle excitation and force distribution at different wall angles and body positions

**DOI:** 10.1007/s00421-025-06047-y

**Published:** 2025-12-19

**Authors:** Eleonora Bonelli, Susanna Rampichini, Fabio Esposito, Giulio Rossi, Alessandro Colombo, Eloisa Limonta

**Affiliations:** 1 Department of Electronics, Information and Bioengineering, Politecnico di Milano, Milan, Italy; 2 Department of Biomedical Sciences for Health, Università degli Studi di Milano, Milan, Italy; 3 ASST Valtellina, Sport Medicine, Eugenio Morelli Hospital, Sondalo, Sondrio Italy

**Keywords:** Rock climbing, Contact forces, Triaxial sensorized hold, Neuromuscular excitation, Surface electromyography

## Abstract

**Purpose:**

In the domain of rock climbing research, existing biomechanical and physiological studies mostly focus on few, typically upper-body muscles and frequently do not replicate ecological conditions. This study investigates full-body muscle excitation and contact forces between climber and holds in two stationary climbing stances.

**Methods:**

Surface electromyography of 22 upper and lower-body muscles and contact forces between climber and holds were recorded in 14 climbers (6 males, 8 females; age 24 ± 6 years; height 1.71 ± 0.07 m; mass 64 ± 8 kg; IRCRA 11–23). We recorded contact forces at three vertices of a rectangle: feet on the lower vertices, right hand on the upper right vertex. The protocol involved five repetitions of two 10-s positions (UP and DOWN) at three wall angles (SLAB: + 5°; VERTICAL: 0°; OVERHANG: −5). Root-mean-square values of electromyographic signals were determined for each repetition and scenario. A two-way RM-ANOVA assessed differences by wall angle and position.

**Results and conclusion:**

Steeper wall angles cause load transfer from feet to hand, homolateral in UP and contralateral in DOWN. DOWN position involves greater excitation of finger flexors muscles and brachioradialis at all wall angles, suggesting that preference for DOWN position in OVERHANG may not be simply explained by the degree of excitation of those muscles. This study provides a comprehensive mapping of full-body superficial muscle excitation and contact forces across two ecological climbing positions at different wall angles, helping to clarify climbing kinetics, inform targeted training and rehabilitation exercises, and optimize protocols for future research.

**Supplementary Information:**

The online version contains supplementary material available at 10.1007/s00421-025-06047-y.

## Introduction

Rock climbing is a rapidly growing sporting activity. As it grows in popularity, a deeper understanding of its biomechanics and physiological demands becomes increasingly valuable. Investigating the principles underlying climbing movements, such as grip forces, body positioning, load distribution, and the corresponding muscular excitation, may aid in optimizing performance through the development of targeted training protocol, and could as well play a crucial role in injury prevention. Yet, the state of the art of rock climbing sciences is still somewhat primitive when compared to other sports. This may be at least partly attributed to the complexity and variability of the involved movements. Some attempts have been made at characterizing climbing movement (Richter et al. 2020), typically through motion capture (Breen et al. 2022; Cha et al. 2015; Iguma et al. 2020; Orth et al. 2017; Pandurevic et al. 2022; Sibella et al. 2007; White and Olsen 2010) or by measuring the interaction forces between climber and holds through load cells (Colombo et al. 2021; Fuss and Niegl 2010, 2008; Lechner et al. 2013; Noé et al. 2001; Quaine and Martin 1999). Other studies investigated muscular strength and fatigue by using ergometers (Esposito et al. 2009; Limonta et al. 2016, 2008; Vigouroux et al. 2015), or explored the activation of selected upper-body muscles during climbing-related exercises (Deyhle et al. 2015; Dykes et al. 2019; Exel et al. 2024; Koukoubis et al. 1995; Kwong et al. 2024; MacLean and Dickerson 2019; Mally et al. 2013; Pühringer et al. 2017). Overall, these studies highlighted the existence of relevant muscular differences between climbers and non-climbers (Esposito et al. 2009; Kwong et al. 2024; Limonta et al. 2016, 2008; Vigouroux et al. 2015), and investigated the fatigue process and muscular excitation involved in some climbing-related exercises (Deyhle et al. 2015; Exel et al. 2024; Mally et al. 2013; Pühringer et al. 2017).

However, with the exception of Deyhle et al. (2015), Mally et al. (2013), and Pühringer et al. (2017), these studies did not involve tasks on a climbing wall. Moreover, all of them examined muscle excitation exclusively in the upper limb musculature, frequently focusing on the forearm muscles (Dykes et al. 2019; Esposito et al. 2009; Kwong et al. 2024; Limonta et al. 2008; Vigouroux et al. 2015). In contrast, in the present study, we investigate the excitation of the main upper- and lower-body superficial muscles involved in the control and stabilization of a full-body climbing task, and the corresponding contact forces with the climbing holds, during two climbing positions representative of the stance typically held when clipping or when resting an arm. The task is executed on a climbing wall at different wall inclinations.

The findings of the present study contribute to: i) clarifying the contact forces expressed when holding a flexed vs extended arm position during observation (when the athlete is planning the next moves), clipping (when the athlete is clipping a quickdraw to a bolt), or active resting and ii) helping to develop a comprehensive map of muscle excitation, identifying which muscles are predominantly engaged, how they contribute to supporting body weight, and evaluating to which extent their involvement changes across the wall angles.

A better understanding of the above aspects may help to explain the athletes’ perceived efficacy of one or the other pose (flexed vs extended arm) and may support the design of more informed training practices. Such a mapping would facilitate the design of future studies on muscular and electromyographic activity during climbing by allowing simplified protocols and a reduced number of sensors without compromising the quality of the data. Lastly, a quantitative understanding of the relation between pose, muscular excitation and loads may help in assessing exercise appropriateness, whether in a rehabilitation context or when planning climbing exercises. Despite the apparent simplicity of the addressed climbing scenario, this study is, to the best of our knowledge, the most comprehensive mapping of superficial muscular excitations and contact forces in an ecological climbing condition.

Given that strength and endurance capacity of the finger flexor and elbow flexor muscles is recognized as key limiting factor in climbing performance (Deyhle et al. 2015; Saul et al. 2019; Stien et al. 2021; Torr et al. 2022), we expect these muscles to be in the set of the most activated ones, along with significant excitation of the shoulder muscles, which play a crucial role in stabilizing the shoulder itself. Additionally, climbers predominantly adopt the flexed arm position on non-overhanging walls, whereas the extended arm position is more frequently used on overhanging walls (Baláš et al. 2017; Exel et al. 2023; Pühringer et al. 2017; Yang et al. 2014). Bearing this in mind, we expect that the perceived advantage of one or the other position be reflected in lower excitation of the finger flexor and elbow flexor muscles in the flexed arm position on non-overhanging walls and a lower excitation in the extended arm position on overhanging walls. We also expect a load transfer from the feet to the hand when analyzing steeper walls, and when comparing the flexed arm position with the extended arm position, since in the flexed arm position body weight is primarily supported by the skeletal structure of the lower limbs, in the extended arm position relies more heavily on the upper limb to support body weight.

## Materials and methods

### Participants

Twenty climbers participated in the study. Inclusion criteria were a prior climbing experience of at least one year, a level of climbing expertise of at least 11 in the International Rock Climbing Research Association (IRCRA) scale, and the absence of any injury in the previous year. All the participants were able to successfully complete the study protocol, but six participants were excluded due to technical issues in the data acquisition. Hence, the remaining dataset is based on 14 participants (six males and eight females, age 24 ± 6 years, height 1.71 ± 0.07 m, body mass 64 ± 8 kg). In detail, three males and six females were at the Intermediate level (11–17 for males and 11–14 for females in the IRCRA scale), and three males and two females were at the Advanced level (18–23 for males and 15–20 for females in the IRCRA scale). All the participants were right-handed.

Participants were informed about the aims of the study, the experimental protocol and the treatment of personal data and signed a declaration of consent prior to the experimental acquisitions accordingly with the latest version of the Declaration of Helsinki. The experimental protocol received approval from the ethical committee of the Politecnico di Milano (approval 38/2023).

### Experimental design

The experimentation was performed on a 2.44 m high and 1.22 m wide indoor climbing wall, with a wall angle adjustable from -10 degrees to + 10 degrees from vertical. The wall was equipped with sensorized hand and footholds, which could be placed on a regular grid spaced 18 cm vertically and 23 cm horizontally. Details about the climbing wall and holds are reported in Sect. “Experimental protocol”.

Neuromuscular excitation and contact forces were recorded while participants assumed two distinct positions (UP, DOWN) on the climbing wall. Data collection was repeated across three different wall angles (SLAB, VERTICAL, OVERHANG), resulting in a total of six scenarios. Details of the positions and wall angles are described in Sect. “Experimental protocol”.

### Experimental protocol

**Fig. 1:**
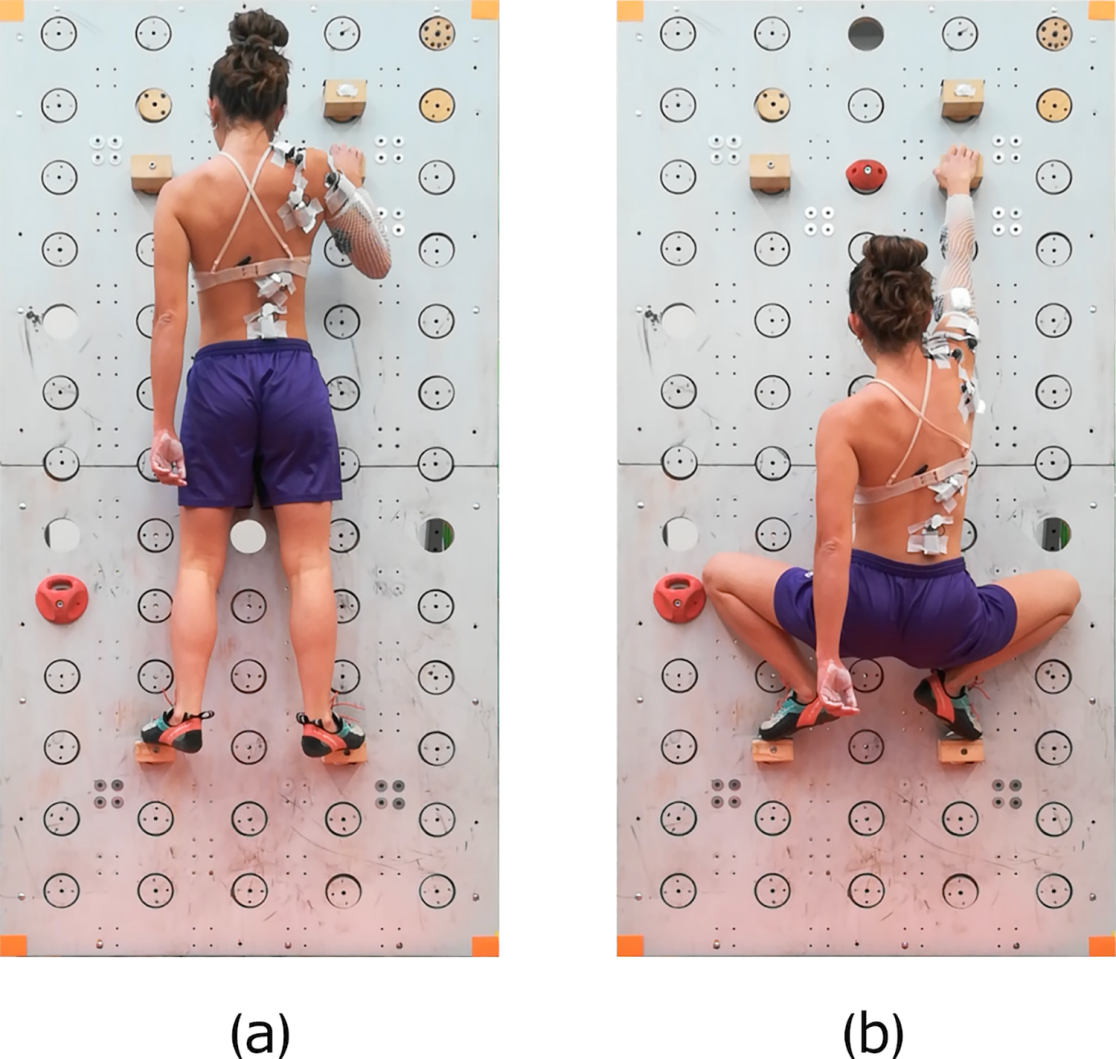
Two different positions performed in the experimental study: **a** UP; **b** DOWN

Upon arrival at the laboratory, participants were informed about the experimental protocol and familiarized with the sensorized wall and the two positions they would be required to assume. In both positions, the holds were placed at three vertices of a rectangle. One position involved fully extended legs and a flexed dominant arm (UP), while the other required bent legs and an extended dominant arm (DOWN). In UP position, the non-dominant arm rested along the body side, whereas in DOWN position it was positioned either in front of or behind the trunk, without leaning on the legs (Fig. 1). These two positions were evaluated at different wall angles from vertical: i) SLAB (+ 5° from vertical, leaning away from the climber, forming an obtuse angle with the ground); ii) VERTICAL (0°); and iii) OVERHANG (-5°, leaning toward the climber, forming an acute angle with the ground). Combinations of the two positions and the three wall angles identified six possible scenarios: UP-SLAB, UP-VERTICAL, UP-OVERHANG, DOWN-SLAB, DOWN-VERTICAL, DOWN-OVERHANG.

Participants were first interviewed about their climbing experience and level, age and height, while body weight was measured immediately before they began climbing to ensure accurate measurements. Then, they were prepared for sEMG acquisition. The skin above the muscles of interest was shaved and gently abraded by an abrasive cream (Nuprep, Weaver and Co., CO, USA) to reduce the impedance at the skin–electrode interface. The sEMG signals were acquired using Ag/AgCl electrodes (active area: 1 cm^2^; ARBO Kendall, Covidien LLC, Mansfield MA, USA), applied with an interelectrode distance of 20 mm. Wireless, bipolar probes (Free-EMG, BTS Bioengineering, Italy) were used to collect the EMG at a sampling frequency of 1 kHz. Since we only had 16 sEMG sensors to acquire 22 muscles, the whole acquisition sequence was repeated twice, with two different sets of muscles, and a 30-min resting time between sequences. The first set of acquisitions concerned the following muscles of the upper limbs and the trunk: *Trapezius* (TRAP); *Anterior Deltoid* (AD); *Posterior Deltoid* (PD); *Pectoralis Major* (PM); *Infraspinatus* (ISPIN); *Latissimus Dorsi* (LD); *Erector Spina*e (ES); *Biceps Brachii* (BB); *Triceps Brachii* (TB); *Brachioradialis* (BRAD); *Flexor Ulnaris* (FULN); *Flexor Radialis* (FRAD). The second set regarded muscles of the lower limb of both sides: *Left Gluteus Maximum* (LGM); *Right Gluteus Maximum* (RGM); *Left Rectus Femoris* (LRF); *Right Rectus Femoris* (RRF); *Left Biceps Femoris* (LBF); *Right Biceps Femoris* (RBF); *Left Gastrocnemius Medialis* (LGAM); *Right Gastrocnemius Medialis* (RGAM); *Left Tibialis Anterior* (LTA); and *Right Tibialis Anterior* (RTA). For each muscle, the sEMG electrodes were placed over the muscle belly according to the recommendations given by Barbero and colleagues (Barbero et al. 2012).

At the beginning of each muscle set acquisition, two repetitions of the maximum voluntary contraction were performed for each investigated muscle to obtain a reference excitation value (maximum voluntary excitation, MVE). The tests for measuring the EMG signal during maximum voluntary contraction of each investigated muscle were conducted by isolating the muscle action according to the recommendations reported by Kendall et al. (1983). Each MVE assessment was preceded by a warm-up sequence consisting of 3–5 contractions at increasing intensity. After completing all the MVEs, participants rested for 10 min, then were asked to approach the climbing wall and tap three times with the right hand on one of the footholds to synchronize the different signal sources. Then, each acquisition included five repetitions of the same sequence consisting of 10 s of UP, 10 s of DOWN (consecutively, without coming down from the wall), and 30 s of recovery time on the ground. The first acquisition in each set always concerned OVERHANG, while the order of VERTICAL and SLAB was randomly assigned. A 5-min resting time was given when transitioning between OVERHANG, VERTICAL, or SLAB, to ensure the complete phosphocreatine recovery kinetic (Layec et al. 2013). OVERHANG was always executed first because, during preliminary trials, participants perceived it as more demanding than both VERTICAL and SLAB, likely because in this scenario the body weight is more heavily supported by the upper limbs. Scheduling the OVERHANG session first allowed us to maximize the resting time that preceded this condition.

**Fig. 2:**
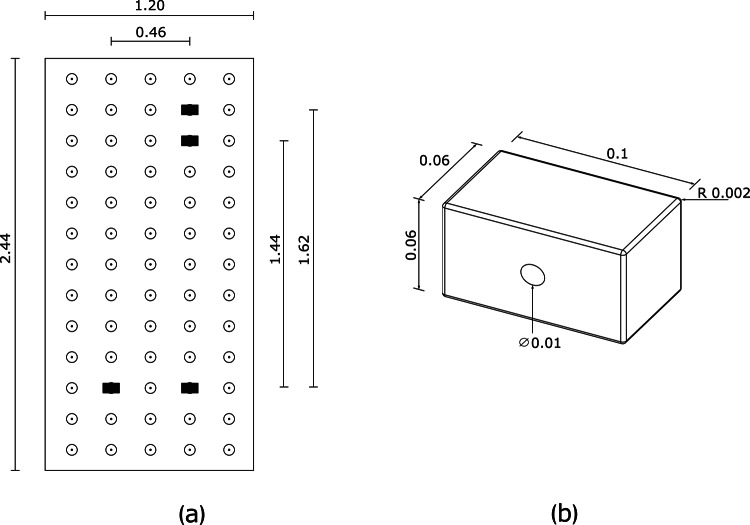
Details of the climbing wall (measures in m): **a** Climbing wall geometry. The black rectangles represent the hold crimps; **b** Hold geometry

Figure 2 shows the climbing wall details, together with holds geometry. Climbing holds were 0.06 m-deep wooden rectangular parallelepipeds with a flat surface covered with abrasive paper to facilitate gripping. The two lower holds served as foot placement and were arranged at a horizontal distance of 0.46 m, while the other hold served as dominant hand placement and was positioned, depending on the height of the participant, either 1.44 m (participant height < 1.75 m) or 1.62 m (participant height ≥ 1.75 m) above the right footholds. Climbers could use all fingers on the surface of the hold and the thumb on the short side (not under and not overlapping the other fingers). The climbing wall and the safety mattress comply with the norm EN 12572-2.

Each climbing hold was equipped with a sensor, hidden within the wall and invisible to the user (Colombo et al. 2023). Each sensor incorporates a triaxial load cell into a hold placement, which measures the magnitude and direction of forces applied to the hold, sampled at 50 Hz. One end of the sensing element is equipped with a wooden disc with an M10 insert that allows the mounting of a standard climbing hold.

**Fig. 3:**
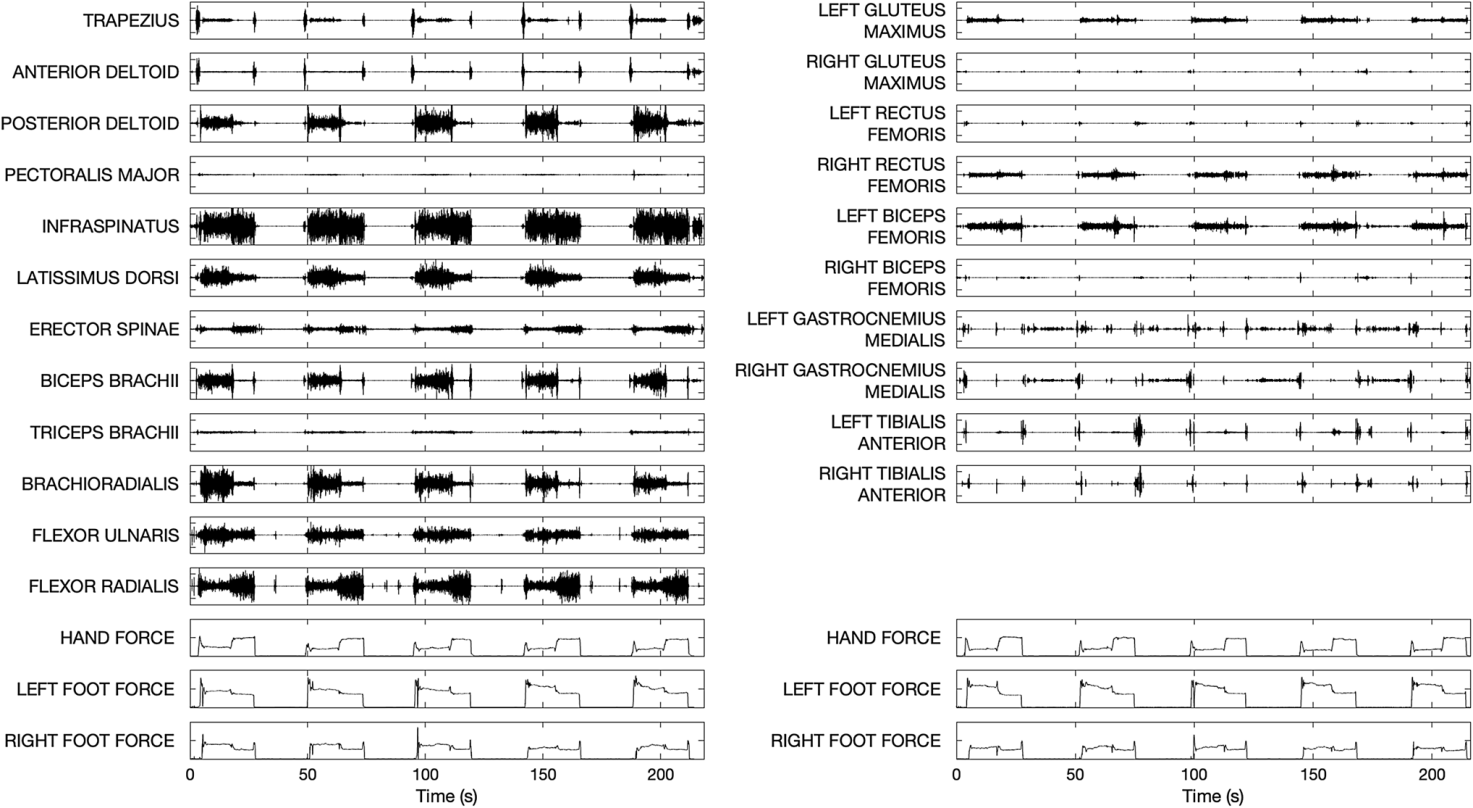
sEMG and force signals from the first and second sets of acquisitions in a representative subject

During each acquisition, 3D-force vectors on the three holds were acquired: the hand force (HAND), the left foot force (LFF), and the right foot force (RFF). Force signals and sEMG signals were simultaneously acquired and synchronized using the inertial unit. sEMG and force signals acquired in a representative subject are shown in Fig. 3, where the left and the right columns refer to the first and second sets of acquisitions, respectively.

### Data analysis

#### Signal preprocessing

The magnitude of the forces on the three holds (HAND, LFF, RFF) was normalized by body weight:

1 $$\left\| {F_{i} } \right\| = \frac{1}{mg}\sqrt {\left( {F_{x} } \right)^{2} + \left( {F_{y} } \right)^{2} + \left( {F_{z} } \right)^{2} }$$

where *i* = {HAND, LFF, RFF}, *m* is the participant’s body mass, and *g* is the gravitational acceleration. Total feet force (TFF) was computed as the sum of RFF and LFF:

2 $$F_{TFF} \, = \,F_{LFF} \, + \,F_{RFF}$$

EMG signal of each muscle was filtered off-line with a zero-lag 4th-order band-pass Butterworth filter (10–400 Hz). Electrocardiogram artifact was removed from PM, LD, ISPIN, and ES signals via basic template subtraction (Petersen et al. 2020).

Force signals were synchronized with sEMG sensors through an EMG-integrated inertial measurement unit (IMU) device (G-Sensor, BTS Bioengineering, Italy) positioned on the right arm, which reliably captured the three peaks corresponding to the initial taps at the beginning of each acquisition. The first peak detected in the signals from both the IMU and the sensorized foothold was used to temporally align the sEMG signals with those from the climbing wall sensors.

We excluded by visual inspection the acquisitions presenting anomalies (e.g., evident acquisition errors). For each participant and each scenario, an expert operator visually inspected the force signals to determine the boundaries [*t*_*start*_*, t*_*stop*_] of the static interval, i.e. the time interval during which the climber was steadily holding the position, for each of the five repetitions. For lack of a well-established best practice, two alternative approaches were used to build the datasets for statistical analysis. The first approach considered the whole static interval selected by the operator. In the second approach, a custom-built algorithm automatically selected a 2-s sub-interval by minimizing the muscle excitation variability within each static interval. In the following, we describe these two approaches, referred to as *whole-interval* and *2 s-interval*, respectively.

##### Whole-interval

Contact forces were computed for each repetition as the mean values of forces computed with Eq. 1 and 2 in [*t*_*start*_*, t*_*stop*_]^*r*^.

3 $$\overline{{F_{i}^{r} }} \, = \,\frac{1}{N}\sum\nolimits_{n = 1}^{N} {\left\| {F_{i,n} } \right\|}$$

where *r* = {1,…,5}, *i* = *{HAND, LFF, RFF}*, *||F*_*i,n*_*||* is the *n*-sample of *i*-force in [*t*_*start*_*, t*_*stop*_]^*r*^ and *N* is the number of samples in [*t*_*start*_*, t*_*stop*_]^*r*^.

Muscle excitation was computed for each repetition as the root mean square (RMS) value, normalized by MVE:

4 $$RMS_{i}^{r} \, = \,\frac{1}{{MVE_{i} }}\sqrt {\frac{1}{N}\sum\nolimits_{n = 1}^{N} {\left| {x_{i,n} } \right|^{2} } }$$

where *r* = {1,…,5}, *i* is the considered muscle, *x*_*i,n*_ is the *n*-sample of *i*-sEMG signal in [*t*_*start*_*, t*_*stop*_]^*r*^ and *N* is the number of samples in [*t*_*start*_*, t*_*stop*_]^*r*^.

##### 2 s-interval

We first computed the RMS series for the whole sEMG signals as in Eq. 4 (epochs of *N* = 1000 samples, overlap 0.9*N*), normalized by MVE. Then, the RMS series were divided in 2-s sub-intervals (*N’* = 2000 samples, overlap 0.9*N’*) within [*t*_*start*_*, t*_*stop*_]^*r*^. For each muscle, the interquartile range (vector *iqr*) was computed as a measure of the variability of muscle excitation. The algorithm selected the 2-s sub-interval *t*_*2s*_ which minimizes the 2-norm of vector *iqr*, defined as:

5 $$iqr_{2} \, = \,\sqrt {\sum\nolimits_{k = 1}^{M} {\left| {iqr_{k} } \right|^{2} } }$$

where *M* is the number of muscles in each set of acquisitions, hence *M* = {12,10} for the first and second sets of acquisitions, respectively. Contact forces were computed for each repetition as in Eq. 3, where *||F*_*i,n*_*||* is the *n*-sample of *i*-force in *t*_*2s*_ and *N* is the number of samples in *t*_*2s*_. Muscle excitation was computed as the mean RMS values in *t*_*2s*_.

Then, the averaged datasets were obtained by computing the mean values of the 10 measures for the contact forces (five repetitions of the two muscle sessions) and of the five measures for the RMS values for each participant and each scenario. In this way, each variable was described by one averaged value for each participant and each scenario.

#### Statistical analysis

Before averaging, a reliability test was conducted on the 10 measures for the contact forces and on the five measures for the RMS values, computing the Intraclass Correlation Coefficient (ICC) for each scenario and each approach. Reliability is defined as *poor* when ICC < 0.5, *moderate* when 0.5 < ICC < 0.75, *good* when 0.75 < ICC < 0.90, *excellent* when ICC > 0.90 (Koo and Li 2016).

A paired Student’s t-test compared the contact forces and *RMS* values obtained through the two approaches (2 s-interval and whole-interval approach). For each approach, two-way ANOVAs for repeated measures (factor1: wall angle; factor2: position) were performed to check for differences in contact forces and muscle excitation among wall angles and between positions. Mauchly’s tests of sphericity were performed beforehand. If the sphericity condition was not met, Greenhouse–Geisser corrections were applied. Pairwise comparisons were computed using Bonferroni corrections. An additional Student’s t-test was performed to compare LFF and RFF for each scenario.

Statistical analyses were performed using IBM SPSS Statistics 29.0.1.0 (IBM Corp., NY, USA), and the significance level was set at α = 0.05.

## Results

Detailed results of the statistical analysis are reported in Supplementary. In the following, only statistically significant results (p < 0.05) are discussed.

### Reliability analysis

Details of the ICC values for each variable and scenario can be found in Table S.1 (Supplementary). Contact forces showed moderate to good reliability (ICC: 0.52–0.88), except for a few cases reported in Table S.1. Muscle excitation showed good to excellent reliability (ICC: 0.75–0.98), except for a few cases reported in Table S.1. Notice that, overall, the whole-interval approach improved reliability.

### Comparison between the 2 s-interval and whole-interval approaches

Contact forces did not show significant differences between the two approaches. In most cases, muscle excitations obtained by the whole-interval approach were slightly higher (mean diff.: 0.001–0.041) compared to the 2 s-interval approach. Details on the significant differences found between the two approaches can be found in Table S.2 (Supplementary).

Despite these differences, the statistical analysis performed on both databases yielded similar findings, except for minimal differences in numerical values. As a consequence, in the following, only the results from the whole-interval dataset are considered.

### Comparison between postures and among wall angles

#### Results on contact forces

Regarding the results of RM-ANOVA, both wall angle and position are significant factors for all contact forces. The combination of wall angle and position is a significant factor for all contact forces, except RFF. See Table S.3 (Supplementary) for details.

**Fig. 4:**
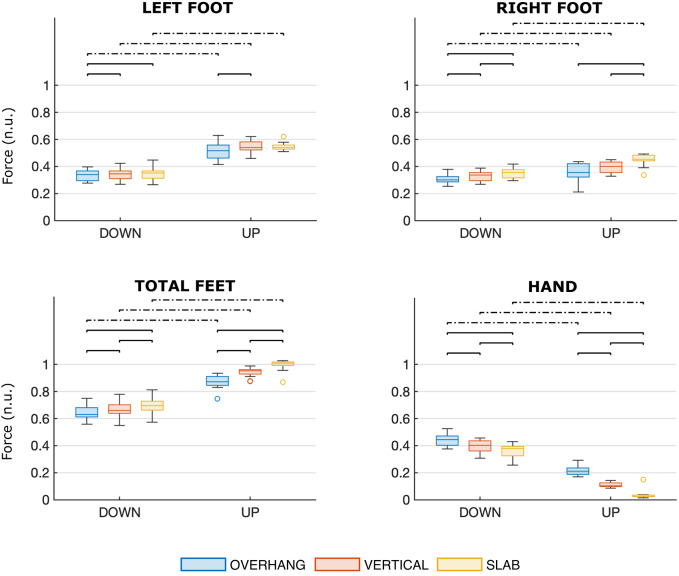
Distributions of the contact forces for each scenario. The colors denote the wall angle (blue for OVERHANG, red for VERTICAL, yellow for SLAB), and the side of the plot denotes the position (DOWN on the left side, STANDING on the right side). Contact forces were normalized to bodyweight and expressed as normalized units (n.u.). Significant differences are marked with a bracket (solid line for significant differences between wall angles, dashed line for significant differences between positions)

Figure 4 shows the distributions of contact forces for each scenario. Significant differences computed with Bonferroni corrections are marked with a bracket. Details of the pairwise comparisons are reported in Tables S.4 and S.5 (Supplementary).

In both positions, the comparison of the load distribution at different wall angles (SLAB, VERTICAL, and OVERHANG) highlights that greater steepness corresponds to a significant increase in HAND (mean diff.: 4–17%, p < 0.001) and a significant decrease in TFF (mean diff.: 3–12%, p < 0.001). LFF and RFF show the same trend, but not all pairwise comparisons are significant.

For all wall angles, the comparison of the load distribution in the two positions (UP and DOWN) highlights that the extended arm position (DOWN) is related to a significant increase in HAND (mean diff.: 23–32%, p < 0.001) and a significant decrease in LFF, RFF, and TFF (mean diff.: 6–30%, p < 0.004). Differences between UP and DOWN are greater in SLAB and progressively decrease in VERTICAL and OVERHANG. Comparing results on LFF (mean diff.: 18–21%, p < 0.001) and RFF (mean diff.: 6–9%, p < 0.004), differences are much greater for LFF.

The t-test performed to compare LFF and RFF in each scenario highlights a significant difference (p < 0.001) in UP-OVERHANG, UP-VERTICAL, and UP-SLAB, where the LFF is significantly higher than RFF. No significant differences are detected in DOWN-OVERHANG, DOWN-VERTICAL, and DOWN-SLAB.

#### Results on muscle excitation

First, we briefly summarize the results of RM-ANOVAs. The wall angle is a significant factor for all muscles (p < 0.021 for all comparisons), except PM, RBF, and RGAM. The position is a significant factor for all muscles, except TRAP, PD, PM, ISPIN, TB, BRAD, RRF, LBF, and RBF. A significant interaction between wall angle and position was observed (p < 0.05 for all interactions) for all muscles, except TRAP, PM, TB, LGM, RGM, RRF, RBF, RGAM, LTA, and RTA. See Table S.3 (Supplementary) for details.

**Fig. 5:**
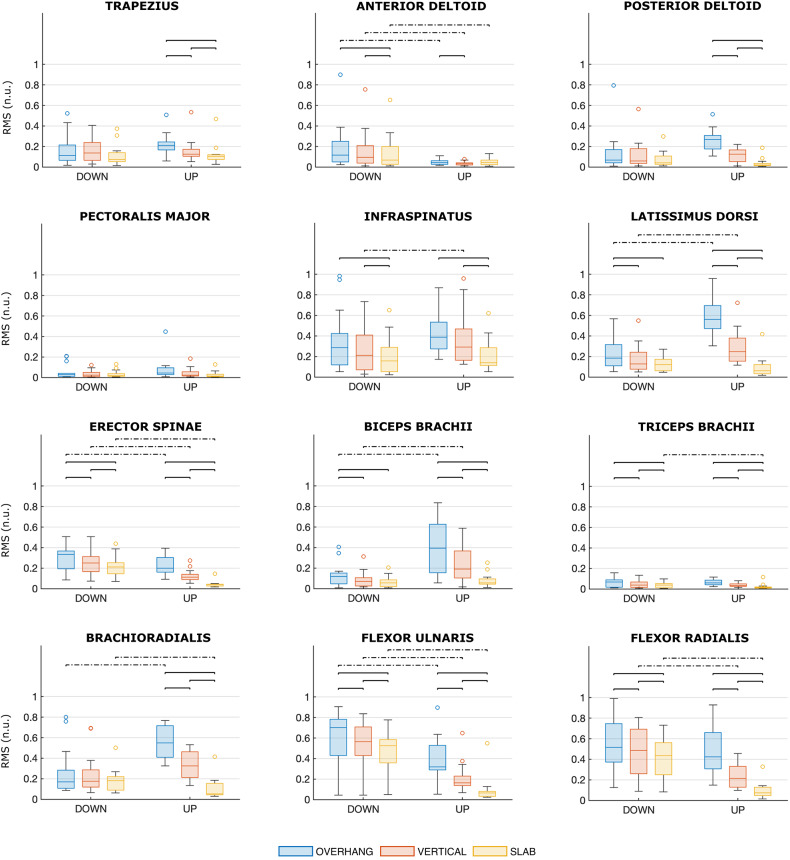
Distributions of the muscle excitations of upper limbs and trunk muscles for each scenario. The colors denote the wall angle (blue for OVERHANG, red for VERTICAL, yellow for SLAB), and the side of the plot denotes the position (DOWN on the left side, STANDING on the right side). Muscle excitation is reported as RMS normalized by the MVE and expressed as normalized units (n.u.). Significant differences are marked with a bracket (solid line for significant differences between wall angles, dashed line for significant differences between positions)

**Fig. 6:**
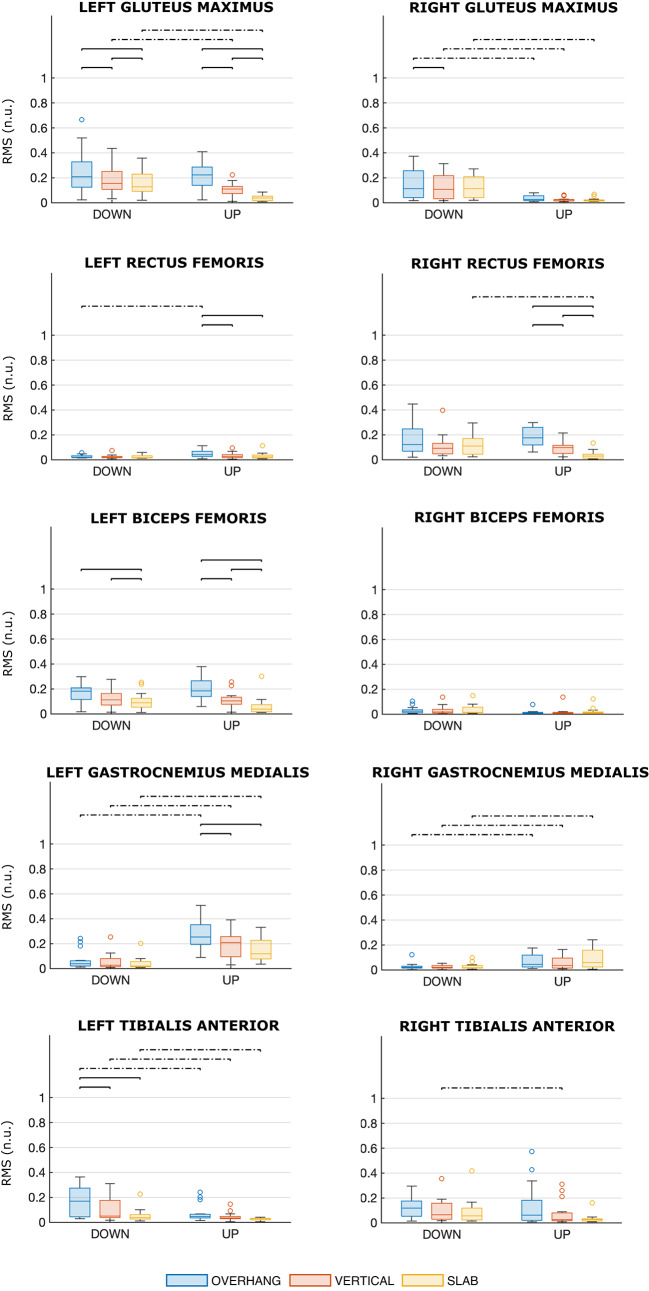
Distributions of the muscle excitations of upper limbs and trunk muscles for each scenario. The colors denote the wall angle (blue for OVERHANG, red for VERTICAL, yellow for SLAB), and the side of the plot denotes the position (DOWN on the left side, STANDING on the right side). Muscle excitation is reported as RMS normalized by the MVE and expressed as normalized units (n.u.). Significant differences are marked with a bracket (solid line for significant differences between wall angles, dashed line for significant differences between positions)

**Fig. 7:**
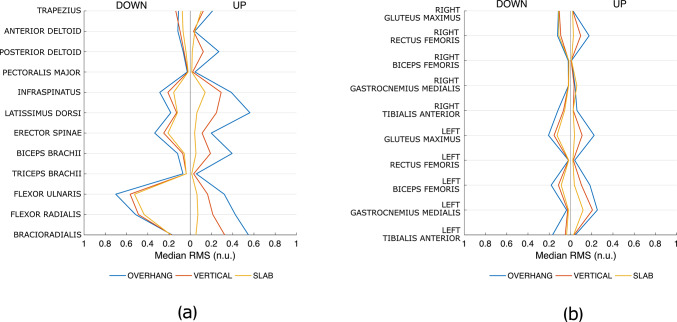
Medians of muscle excitation for each muscle and scenario: **a** Upper limb and trunk muscles; **b** Lower limbs muscles. The colors denote the angle (blue for OVERHANG, red for VERTICAL, yellow for SLAB), and the side of the plot denotes the position (DOWN on the left side, UP on the right side). Muscle excitation is reported as RMS normalized by the MVE and expressed as normalized units (n.u.)

Figures 5 and 6 show the distributions of muscle excitation for upper body muscles and lower limb muscles, respectively. Significant differences between the scenarios computed with Bonferroni corrections are marked with a bracket. For a general evaluation of the average muscle excitation as the scenario changes, Fig. 7 shows that, for almost all investigated muscles, median RMS values in OVERHANG (blue line) are higher than in VERTICAL (red line), which in turn are higher than in SLAB (yellow line).

Table S.4 (Supplementary) reports pairwise comparisons as the wall angle changes. Regarding UP position, a steeper wall corresponds to greater excitation. The difference is more pronounced (mean diff.: 21–51%, p < 0.001) for LD and BRAD. ISPIN, BB, FULN, and FRAD also exhibit a marked increase in muscle excitation (mean diff.: 12–38%, p < 0.005). TRAP, PD, ES, TB show significant differences, but smaller in absolute values (mean diff.: 2–21%, p < 0.038). Speaking of lower limb muscles, the analysis detected a significant increase in excitation (mean diff.: 1–17%, p < 0.025) for LGM, LRF, RRF, LBF, and LGAM, although not all pairwise comparisons showed statistically significant differences (see Fig. 6 for details).

Regarding DOWN position, the trend is similar: a steeper wall is generally associated with greater excitation, but there are fewer significant differences. In particular, BRAD, PD, and TRAP do not exhibit significant differences (as opposed to UP). The significant differences for the other upper body muscles are also smaller in absolute value (mean diff.: 1–16%, p < 0.048). Among the lower limb muscles, there is a significant increase in excitation (mean diff.: 2–10%, p < 0.044) for LGM, RGM, LBF, and LTA.

Table S.5 (Supplementary) reports pairwise comparisons as the position changes. In some cases, muscle excitation is greater in DOWN. The difference is more pronounced (mean diff.: 21–37%, p < 0.014) for FULN, FRAD (SLAB and VERTICAL), and BRAD (OVERHANG). BRAD (SLAB), ES, and AD show the same trend, but the difference is smaller in absolute value (mean diff.: 7–18%, p < 0.043). In a few cases, we observe greater muscle excitation in UP (mean diff.: 12–36%, p < 0.033): ISPIN (VERTICAL), LD (VERTICAL and OVERHANG), and BB (VERTICAL and OVERHANG). Summarizing, the finger flexor muscles show greater excitation in DOWN (FULN at all wall angles, FRAD in VERTICAL and SLAB), while muscle excitation appears greater in UP for ISPIN (OVERHANG) and LD and BB (OVERHANG and VERTICAL). Speaking of lower limb muscles, the muscle excitation is greater in UP (mean diff.: 2–19%, p < 0.032) for LGAM, RGAM, and LRF (OVERHANG only), while it is greater in DOWN (mean diff.: 3–12%, p < 0.045) for LGM, RGM, RRF, LTA, and RTA, although not all pairwise comparisons showed statistically significant differences (see Fig. 6 for details).

## Discussion

We investigated two climbing positions at three different wall angles. We acquired contact force measurements through a sensorized climbing wall and muscle excitation measurements from sEMG sensors. In detail, a total of 22 muscles were considered, both from the upper body and lower limbs.

In both UP and DOWN position, the comparison of the load distribution at different wall angles (SLAB, VERTICAL, OVERHANG) highlights a load transfer from the feet to the hand as the wall becomes steeper. The load transfer is predominantly homolateral: most of the load transfers from the right foot to the (right) hand. A load transfer from feet to hand is to be expected, since in SLAB, the centre of gravity falls close to the convex envelope of the contact surfaces of the feet on the footholds. As a consequence, most of the body weight conveys to the lower limbs. In OVERHANG, on the other hand, the centre of gravity is forced to stand farther away from the convex envelope of the contact surfaces of the feet, forcing a greater involvement of the upper limbs and consequently a lower load on the feet. The fact that this load transfer is mainly homolateral was, however, not obvious, and is probably explained by the need to keep sufficient load on the contralateral foot. Indeed, a certain amount of load on the left foot is required to counteract the torque generated by the offset between the body’s center of mass and the axis connecting the right hand and foot, which would rotate the climber around the vector going from the right foot to the right hand. We expect this load transfer to become more evenly distributed between the feet if ground projection of the handhold were to approach the midpoint between the projection of the two footholds.

Comparing the flexed leg position (DOWN) with the extended leg position (UP), we observed both a homolateral and contralateral load transfer. However, the load transfer from the hand to the contralateral foot appeared predominant. Once again, this load transfer aligns with expectations: in UP position, body weight is primarily supported by the skeletal structure of the lower limbs, reducing the upper limb’s contribution, while DOWN position relies more heavily on the upper limb to support body weight. Additionally, the predominance of contralateral load transfer may be explained by the fact that, in DOWN position, the centre of mass can be held closer to the axis between the homolateral foot and hand than in UP position. Additional load on the contralateral foot is therefore needed to compensate for the increasing torque in UP position. Therefore, this difference may be attenuated when the ground projection of the handhold approaches the midpoint between the projections of the two footholds.

For what concerns our results on muscle excitation, our analysis had the double aim of determining which muscles are predominantly involved in holding the two studied positions, and how their involvement changes among the three wall angles.

In UP position, only the infraspinatus presented excitations exceeding 40% of the MVE for all angles; in VERTICAL and OVERHANG, also flexors ulnaris (only OVERHANG) and radialis, brachioradialis, latissimus dorsi, and biceps brachii were excited above this threshold. In DOWN position, the muscles exhibiting excitation levels exceeding 40% of the MVE are flexor ulnaris and radialis at all wall angles, infraspinatus in OVERHANG and VERTICAL, brachioradialis and latissimus dorsi in OVERHANG. A considerable excitation of the finger flexor and elbow flexor muscles, at least in VERTICAL and OVERHANG, was expected as these are known to be the main limiting muscles in performance (Deyhle et al. 2015; Saul et al. 2019; Stien et al. 2021; Torr et al. 2022). The high excitation of infraspinatus and latissimus dorsi highlights that these two muscles are relevant in the kinetics of the studied positions. We should notice that the absence of the lower body muscles in the above list does not necessarily imply their irrelevance in holding the studied positions, but it is more likely due to their higher MVE.

The comparison of the muscle excitation at different wall angle (SLAB, VERTICAL, OVERHANG) highlights that a steeper wall corresponds to greater excitation for most of investigated muscles within each position, consistently with what was observed by Pühringer et al. (2017), and no muscle exhibits significantly lower excitation. Indeed, with decreasing the wall angle (from a slab to a vertical, and then to an overhanging wall), the contribution of the skeletal structure to supporting body weight becomes less relevant. The trend is the same for both upper body muscles and lower limb muscles, but it is much more prominent for upper body muscles. Moreover, the lower limb muscles that exhibit higher excitation in the OVERHANG scenario, compared to SLAB and VERTICAL, in both positions (left gluteus maximus and biceps femoris, right rectus femoris, and marginally left rectus femoris, gastrocnemius medialis, tibialis anterior, and right gluteus maximus) do not contribute to generating a higher load on the feet, as we observed above. This presumably means that these higher excitation serves primarily to stabilize the posture.

Analyzing the pattern of variation of muscle excitation between positions is less straightforward. As we discussed above, comparing DOWN with UP highlighted a load transfer from hand to feet, irrespective of the wall angle. Moreover, our sEMG data shows that, at all wall angles, DOWN is related to a higher excitation of the anterior deltoid, erector spinae, brachioradialis, flexor ulnaris, and radialis compared to UP. This set contains some finger flexor muscles (flexor ulnaris and radialis) and brachioradialis, which are known to be the limiting muscles in climbing (Deyhle et al. 2015; Saul et al. 2019; Stien et al. 2021; Torr et al. 2022). The advantage of DOWN over UP position when resting in OVERHANG cannot, as a consequence, be explained in terms of a lower excitation of the limiting muscles. We also observe that, in OVERHANG, UP position is related to a greater excitation of the latissimus dorsi and the biceps brachii than DOWN position. Hence, the advantage of DOWN over UP position in OVERHANG may result from a rather complex sequence of muscle fatigue buildup and posture adjustments, rather than the degree of excitation of the limiting finger flexor muscles. Understanding such a sequence would however require sEMG and postural data from a fatigue-to-exhaustion experiment, falling out of the scope of this work. Overall, our observation suggests that studies focusing on the forearm muscles (Dykes et al. 2019; Esposito et al. 2009; Kwong et al. 2024; Limonta et al. 2008; Vigouroux et al. 2015) may miss details that are relevant to explaining climbing fatigue, endurance, and performance, and more generally that a comprehensive view of full-body muscular excitation will be necessary to explain the processes involved in executing even the simplest climbing exercises. Additionally, the relative importance of the latissimus dorsii over the finger and elbow flexors muscles (Deyhle et al. 2015) may have to be reconsidered when investigating climbing fatigue and resting positions.

The present study has several limitations that warrant consideration. First, although we varied positions and wall angles to capture a broad range of scenarios, we acknowledge that climbing involves far greater variability than what our experimental design could capture. The joint angles were defined by the investigated and only the dominant hand was used. Second, the wall angles varied between –5° and + 5° from vertical: more extreme inclinations could elicit markedly different neuromuscular and postural strategies. Third, the sample consisted of intermediate and advance climbers but not elite-level climbers. However, we expect that the changes in load distribution and muscle excitation that we observed between the six scenarios will persist and possibly be more pronounced in a population of elite climbers. Fourth, the absence of objective or subjective indicators of fatigue prevented us from monitoring the recovery status and detecting potential residual signs of fatigue, especially after the OVERHANG acquisition. Nonetheless, such an effect, if present, was limited due to the precautions reported in Sect. “Experimental protocol”. Last, we did not perform an inter-operator reliability analysis to assess the effect of the operator’s selection of the signal window for data analysis. However, the fact that we obtain very similar results using the (operator-driven) whole-interval approach and the (algorithmic) 2 s-interval approach suggests that operator’s effect is limited.

Future research could explore these additional possibilities, as well as include the study of dynamic movements expanding beyond the analysis of static stances. Indeed, dynamic component in competitive climbing is becoming increasingly important, involving a highly diverse variety of movements.

## Conclusions

This study gives an accurate quantitative evaluation of load distribution between the limbs as a function of the wall angle, and a complete characterization of the muscular excitation among the main superficial muscles involved in maintaining the two examined positions. In SLAB and VERTICAL, the measured load distribution and muscular excitations show that UP position (flexed arm and extended legs) allows for a greater portion of body weight to be supported by the lower limbs and overall reduces the excitation of upper body muscles with respect to DOWN position (flexed legs and extended arm). This is consistent with the use of UP versus DOWN as a resting or clipping stance when climbing on wall inclinations akin to our SLAB and VERTICAL scenarios. In OVERHANG, on the other hand, the hand is more heavily loaded in DOWN than in UP, and DOWN exhibits a greater excitation of the finger flexor muscles and brachioradialis than UP. This suggests that a focus on the forearm muscles when investigating climbing fatigue, endurance, or performance may provide only partial results. Other muscles (such as latissimus dorsi and biceps brachii) must be involved in explaining the processes related to fatigue build-up and climbing performance.

All the investigated muscles showed significant variation in excitation across positions and wall inclinations, with the exception of pectoralis major and right biceps femoris. Consequently, all the muscles considered in this study (with the above exceptions) presumably play a relevant role in the execution of the tested stances and should be considered when studying the kinetics of related exercises. The degree of variation of muscular excitation however changes across the different scenarios, with brachioradialis, flexors radialis and ulnaris, posterior deltoid, biceps brachii, infraspinatus, and latissimus dorsi, exhibiting the greatest variability, and other muscles exhibiting generally lower variability. The quantitative results reported above allows ranking the contributions of the investigated muscles, thereby enabling the selection of the most relevant subset for a given scenario.

The present work provides a solid quantitative basis for future studies involving more complex climbing movements, including potential investigations into the therapeutic effects of climbing.

## Supplementary Information

Below is the link to the electronic supplementary material.

Supplementary file1 (DOCX 49 KB)

## Data Availability

The data that support the findings of this study are available from the corresponding author, AC, upon reasonable request.

## Abbreviations

AD: Anterior deltoid
BB: Biceps brachii
BRAD: Brachioradialis
ES: Erector spinae
FRAD: Flexor radialis
FULN: Flexor ulnaris
HAND: Hand force
ISPIN: Infraspinatus
LBF: Left biceps femoris
LD: Latissimus dorsi
LFF: Left foot force
LGAM: Left gastrocnemius medialis
LGM: Left gluteus maximus
LRF: Left rectus femoris
LTA: Left tibialis anterior
MVE: Maximum voluntary excitation
PD: Posterior deltoid
PM: Pectoralis major
RBF: Right biceps femoris
RFF: Right foot force
RGAM: Right gastrocnemius medialis
RGM: Right gluteus maximus
RRF: Right rectus femoris
RTA: Right tibialis anterior
sEMG: Surface electromyography
TB: Triceps brachii
TFF: Total feet force
TRAP: Trapezius
