# Supplementary material for: Climber’s muscle excitation and force distribution at different wall angles and body positions
Source: Eur J Appl Physiol. 2025 Dec 19;126(5):2779–93. doi: 10.1007/s00421-025-06047-y (PMC13236747; doi:10.1007/s00421-025-06047-y)
Supplement: Supplementary file 1 — Supplementary file1 (DOCX 49 KB) [file 421_2025_6047_MOESM1_ESM.docx]

Table S.1: Results of reliability analysis conducted on the 10 measures for the contact forces and on the five measures for the RMS values computing the Intraclass Correlation Coefficient (ICC) for each scenario and each approach. Reliability is defined *poor* when ICC<0.5, *moderate* when 0.5<ICC<0.75, *good* when 0.75<ICC<0.90, *excellent* when ICC>0.90.

| **Variable** | **Approach** | **UP** | | | **DOWN** | | |
| --- | --- | --- | --- | --- | --- | --- | --- |
|  |  | **OVERHANG** | **VERTICAL** | **SLAB** | **OVERHANG** | **VERTICAL** | **SLAB** |
| HAND | 2s-interval | 0.61 | 0.49 | 0.85 | 0.63 | 0.73 | 0.71 |
|  | whole-interval | 0.62 | 0.52 | 0.86 | 0.66 | 0.75 | 0.73 |
| LF | 2s-interval | 0.49 | 0.47 | 0.44 | 0.72 | 0.80 | 0.87 |
|  | whole-interval | 0.53 | 0.52 | 0.49 | 0.73 | 0.83 | 0.88 |
| RF | 2s-interval | 0.61 | 0.49 | 0.68 | 0.62 | 0.73 | 0.69 |
|  | whole-interval | 0.65 | 0.53 | 0.76 | 0.64 | 0.72 | 0.70 |
| TF | 2s-interval | 0.67 | 0.66 | 0.84 | 0.68 | 0.79 | 0.76 |
|  | whole-interval | 0.69 | 0.70 | 0.86 | 0.71 | 0.80 | 0.77 |
| TRAP | 2s-interval | 0.90 | 0.86 | 0.87 | 0.72 | 0.72 | 0.74 |
|  | whole-interval | 0.93 | 0.88 | 0.91 | 0.80 | 0.76 | 0.85 |
| AD | 2s-interval | 0.86 | 0.62 | 0.77 | 0.91 | 0.92 | 0.96 |
|  | whole-interval | 0.92 | 0.74 | 0.83 | 0.94 | 0.95 | 0.97 |
| PD | 2s-interval | 0.65 | 0.82 | 0.90 | 0.69 | 0.88 | 0.81 |
|  | whole-interval | 0.83 | 0.83 | 0.94 | 0.79 | 0.88 | 0.79 |
| PM | 2s-interval | 0.98 | 0.96 | 0.97 | 0.94 | 0.97 | 0.97 |
|  | whole-interval | 0.97 | 0.88 | 0.98 | 0.79 | 0.75 | 0.85 |
| ISPIN | 2s-interval | 0.77 | 0.94 | 0.77 | 0.91 | 0.89 | 0.93 |
|  | whole-interval | 0.79 | 0.95 | 0.81 | 0.90 | 0.91 | 0.92 |
| LD | 2s-interval | 0.85 | 0.88 | 0.86 | 0.88 | 0.88 | 0.80 |
|  | whole-interval | 0.88 | 0.90 | 0.87 | 0.88 | 0.93 | 0.82 |
| ES | 2s-interval | 0.93 | 0.86 | 0.79 | 0.85 | 0.92 | 0.88 |
|  | whole-interval | 0.95 | 0.88 | 0.84 | 0.91 | 0.92 | 0.91 |
| BB | 2s-interval | 0.85 | 0.83 | 0.64 | 0.90 | 0.84 | 0.67 |
|  | whole-interval | 0.90 | 0.88 | 0.78 | 0.93 | 0.81 | 0.82 |
| TB | 2s-interval | 0.76 | 0.84 | 0.86 | 0.81 | 0.81 | 0.85 |
|  | whole-interval | 0.89 | 0.90 | 0.92 | 0.88 | 0.88 | 0.90 |
| BRAD | 2s-interval | 0.46 | 0.75 | 0.90 | 0.86 | 0.93 | 0.87 |
|  | whole-interval | 0.50 | 0.85 | 0.94 | 0.90 | 0.91 | 0.90 |
| FULN | 2s-interval | 0.80 | 0.76 | 0.95 | 0.96 | 0.97 | 0.92 |
|  | whole-interval | 0.89 | 0.80 | 0.96 | 0.97 | 0.97 | 0.95 |
| FRAD | 2s-interval | 0.89 | 0.82 | 0.90 | 0.95 | 0.95 | 0.91 |
|  | whole-interval | 0.92 | 0.83 | 0.90 | 0.96 | 0.98 | 0.94 |
| LGM | 2s-interval | 0.86 | 0.89 | 0.73 | 0.86 | 0.84 | 0.86 |
|  | whole-interval | 0.88 | 0.88 | 0.74 | 0.87 | 0.81 | 0.86 |
| RGM | 2s-interval | 0.83 | 0.91 | 0.83 | 0.82 | 0.91 | 0.79 |
|  | whole-interval | 0.68 | 0.91 | 0.76 | 0.84 | 0.86 | 0.77 |
| LRF | 2s-interval | 0.82 | 0.90 | 0.87 | 0.82 | 0.86 | 0.79 |
|  | whole-interval | 0.89 | 0.94 | 0.91 | 0.67 | 0.83 | 0.81 |
| RRF | 2s-interval | 0.83 | 0.65 | 0.64 | 0.69 | 0.76 | 0.88 |
|  | whole-interval | 0.79 | 0.74 | 0.78 | 0.74 | 0.76 | 0.88 |
| LBF | 2s-interval | 0.93 | 0.90 | 0.90 | 0.90 | 0.93 | 0.91 |
|  | whole-interval | 0.92 | 0.93 | 0.95 | 0.86 | 0.94 | 0.92 |
| RBF | 2s-interval | 0.54 | 0.95 | 0.83 | 0.81 | 0.90 | 0.93 |
|  | whole-interval | 0.66 | 0.96 | 0.86 | 0.89 | 0.91 | 0.89 |
| LGAM | 2s-interval | 0.81 | 0.80 | 0.88 | 0.89 | 0.85 | 0.94 |
|  | whole-interval | 0.80 | 0.85 | 0.86 | 0.92 | 0.88 | 0.94 |
| RGAM | 2s-interval | 0.87 | 0.85 | 0.87 | 0.70 | 0.41 | 0.61 |
|  | whole-interval | 0.84 | 0.84 | 0.87 | 0.61 | 0.55 | 0.68 |
| LTA | 2s-interval | 0.85 | 0.37 | 0.73 | 0.84 | 0.78 | 0.67 |
|  | whole-interval | 0.86 | 0.57 | 0.66 | 0.84 | 0.82 | 0.74 |
| RTA | 2s-interval | 0.90 | 0.91 | 0.60 | 0.71 | 0.92 | 0.85 |
|  | whole-interval | 0.90 | 0.87 | 0.75 | 0.71 | 0.88 | 0.89 |

Table S.2: Results of paired t-test performed to compare the datasets from the two approaches. Each cell reports the t-statistics on the first line, the p-value on the second line, and the mean difference (standard deviation) on the third line. Statistically significant results are highlighted in bold.

| **Variable** | **UP** | | | **DOWN** | | |
| --- | --- | --- | --- | --- | --- | --- |
|  | **OVERHANG** | **VERTICAL** | **SLAB** | **OVERHANG** | **VERTICAL** | **SLAB** |
| HAND | t(14) = 0.330 | t(15) = 1.832 | t(14) = 1.316 | t(14) = -0.367 | t(15) = -1.266 | t(14) = -1.627 |
|  | p = 0.746 | p = 0.087 | p = 0.209 | p = 0.719 | p = 0.225 | p = 0.126 |
|  | <0.001 (0.003) | 0.001 (0.002) | 0.001 (0.002) | <0.001 (0.002) | -0.001 (0.003) | -0.001 (0.003) |
| LF | t(14) = 0.152 | t(15) = 0.328 | t(14) = 0.590 | t(14) = 0.804 | t(15) = 0.064 | t(14) = 0.810 |
|  | p = 0.881 | p = 0.748 | p = 0.565 | p = 0.435 | p = 0.950 | p = 0.432 |
|  | <0.001 (0.007) | <0.001 (0.003) | <0.001 (0.002) | <0.001 (0.002) | <0.001 (0.002) | <0.001 (0.002) |
| RF | t(14) = -0.636 | t(15) = -1.215 | t(14) = -0.513 | t(14) = 0.522 | t(15) = 1.999 | t(14) = 1.407 |
|  | p = 0.535 | p = 0.243 | p = 0.616 | p = 0.610 | p = 0.064 | p = 0.181 |
|  | -0.001 (0.008) | -0.001 (0.004) | <0.001 (0.004) | <0.001 (0.002) | 0.002 (0.003) | 0.001 (0.003) |
| TF | t(14) = -1.292 | t(15) = -1.500 | t(14) = -0.317 | t(14) = 0.912 | t(15) = 1.735 | t(14) = 2.103 |
|  | p = 0.217 | p = 0.154 | p = 0.756 | p = 0.377 | p = 0.103 | p = 0.054 |
|  | -0.001 (0.003) | -0.001 (0.003) | <0.001 (0.002) | 0.001 (0.003) | 0.002 (0.004) | 0.001 (0.003) |
| TRAP | t(14) = 1.672 | **t(15) = 3.847** | **t(14) = 3.710** | **t(14) = 2.482** | **t(15) = 2.964** | **t(14) = 2.340** |
|  | p = 0.117 | **p = 0.002** | **p = 0.002** | **p = 0.026** | **p = 0.010** | **p = 0.035** |
|  | 0.005 (0.012) | **0.010 (0.011)** | **0.008 (0.008)** | **0.013 (0.020)** | **0.013 (0.017)** | **0.017 (0.029)** |
| AD | **t(14) = 2.460** | t(15) = 1.999 | **t(14) = 3.218** | **t(14) = 2.918** | **t(15) = 3.353** | t(14) = 1.797 |
|  | **p = 0.028** | p = 0.064 | **p = 0.006** | **p = 0.011** | **p = 0.004** | p = 0.094 |
|  | **0.002 (0.003)** | 0.002 (0.003) | **0.004 (0.005)** | **0.019 (0.026)** | **0.019 (0.022)** | 0.008 (0.017) |
| PD | t(14) = 0.098 | **t(15) = 4.173** | t(14) = 0.498 | t(14) = 0.943 | t(15) = 1.936 | t(14) = 0.950 |
|  | p = 0.923 | **p<0.001** | p = 0.626 | p = 0.362 | p = 0.072 | p = 0.358 |
|  | 0.001 (0.035) | **0.005 (0.005)** | 0.001 (0.004) | 0.011 (0.047) | 0.015 (0.031) | 0.010 (0.040) |
| PM | t(14) = -0.998 | t(15) = -0.972 | t(14) = -0.975 | t(14) = -0.929 | t(15) = -0.873 | t(14) = -0.769 |
|  | p = 0.335 | p = 0.347 | p = 0.346 | p = 0.369 | p = 0.397 | p = 0.455 |
|  | -0.144 (0.558) | -0.053 (0.219) | -0.010 (0.040) | -0.069 (0.287) | -0.035 (0.160) | -0.023 (0.116) |
| ISPIN | **t(14) = 2.475** | **t(15) = 2.494** | **t(14) = 2.890** | t(14) = 1.966 | **t(15) = 2.542** | **t(14) = 3.713** |
|  | **p = 0.027** | **p = 0.025** | **p = 0.012** | p = 0.069 | **p = 0.023** | **p = 0.002** |
|  | **0.028 (0.044)** | **0.023 (0.038)** | **0.010 (0.014)** | 0.012 (0.024) | **0.013 (0.021)** | **0.015 (0.016)** |
| LD | **t(14) = 3.462** | t(15) = 1.949 | **t(14) = 3.589** | t(14) = 1.093 | **t(15) = 2.326** | t(14) = 1.955 |
|  | **p = 0.004** | p = 0.070 | **p = 0.003** | p = 0.293 | **p = 0.034** | p = 0.071 |
|  | **0.019 (0.021)** | 0.008 (0.016) | **0.007 (0.008)** | 0.002 (0.008) | **0.009 (0.015)** | 0.007 (0.013) |
| ES | t(14) = 1.278 | **t(15) = 2.240** | **t(14) = 5.920** | **t(14) = 3.751** | **t(15) = 3.356** | **t(14) = 2.810** |
|  | p = 0.222 | **p = 0.041** | **p<0.001** | **p = 0.002** | **p = 0.004** | **p = 0.014** |
|  | 0.002 (0.006) | **0.003 (0.006)** | **0.003 (0.002)** | **0.008 (0.008)** | **0.007 (0.009)** | **0.006 (0.002)** |
| BB | **t(14) = 3.044** | **t(15) = 3.019** | **t(14) = 2.382** | **t(14) = 3.126** | t(15) = 1.340 | t(14) = 2.126 |
|  | **p = 0.009** | **p = 0.009** | **p = 0.032** | **p = 0.007** | p = 0.200 | p = 0.052 |
|  | **0.018 (0.023)** | **0.011 (0.014)** | **0.005 (0.007)** | **0.013 (0.015)** | 0.006 (0.019) | 0.006 (0.010) |
| TB | t(14) = 0.778 | **t(15) = 3.095** | **t(14)=2.304** | **t(14) = 3.034** | **t(15) = 2.584** | t(14) = 0.951 |
|  | p = 0.450 | **p = 0.007** | **p = 0.037** | **p = 0.009** | **p = 0.021** | p = 0.358 |
|  | 0.001 (0.003) | **0.002 (0.003)** | **0.002 (0.003)** | **0.007 (0.008)** | **0.004 (0.006)** | 0.002 (0.006) |
| BRAD | **t(14) = 3.677** | **t(15) = 3.228** | t(14) = 1.960 | **t(14) = 2.352** | t(15) = 1.451 | t(14) = 1.601 |
|  | **p = 0.002** | **p = 0.006** | p = 0.070 | **p = 0.034** | p = 0.167 | p = 0.132 |
|  | **0.024 (0.025)** | **0.016 (0.020)** | 0.014 (0.028) | **0.011 (0.018)** | 0.008 (0.021) | 0.012 (0.030) |
| FULN | **t(14) = 2.643** | **t(15) = 5.197** | **t(14) = 2.558** | **t(14) = 2.543** | t(15) = 0.247 | t(14) = -0.752 |
|  | **p = 0.019** | **p<0.001** | **p = 0.023** | **p = 0.023** | p = | p = 0.464 |
|  | **0.014 (0.021)** | **0.013 (0.010)** | **0.019 (0.028)** | **0.010 (0.015)** | 0.001 (0.019) | -0.003 (0.014) |
| FRAD | **t(14) = 2.426** | **t(15) = 3.081** | **t(14) = 2.729** | **t(14) = 2.235** | t(15) = 1.133 | **t(14) = 2.238** |
|  | **p = 0.029** | **p = 0.008** | **p = 0.016** | **p = 0.042** | p = 0.275 | **p = 0.042** |
|  | **0.027 (0.044)** | **0.022 (0.028)** | **0.021 (0.029)** | **0.017 (0.030)** | 0.006 (0.023) | **0.013 (0.022)** |
| LGM | t(14) = 1.979 | **t(15) = 2.488** | **t(14) = 2.829** | **t(14) = 4.459** | **t(15) = 3.723** | **t(14) = 5.768** |
|  | p = 0.068 | **p = 0.025** | **p = 0.013** | **p<0.001** | **p = 0.002** | **p<0.001** |
|  | 0.006 (0.012) | **0.004 (0.007)** | **0.002 (0.003)** | **0.026 (0.022)** | **0.022 (0.023)** | **0.016 (0.011)** |
| RGM | t(14) = 1.068 | **t(15) = 3.201** | **t(14) = 2.807** | **t(14) = 3.911** | **t(15) = 3.192** | **t(14) = 3.223** |
|  | p = 0.303 | **p = 0.006** | **p = 0.014** | **p = 0.002** | **p = 0.006** | **p = 0.006** |
|  | 0.003 (0.010) | **0.002 (0.002)** | **0.002 (0.003)** | **0.016 (0.016)** | **0.029 (0.037)** | **0.020 (0.024)** |
| LRF | **t(14) = 3.856** | **t(15) = 3.038** | **t(14) = 3.139** | **t(14) = 3.115** | **t(15) = 3.461** | **t(14) = 3.508** |
|  | **p = 0.002** | **p = 0.008** | **p = 0.007** | **p = 0.008** | **p = 0.003** | **p = 0.003** |
|  | **0.008 (0.008)** | **0.005 (0.007)** | **0.007 (0.009)** | **0.003 (0.004)** | **0.002 (0.003)** | **0.002 (0.002)** |
| RRF | **t(14) = 3.276** | **t(15) = 3.624** | **t(14) = 2.768** | t(14) = 1.885 | **t(15) = 5.205** | **t(14) = 3.165** |
|  | **p = 0.006** | **p = 0.003** | **p = 0.015** | p = 0.080 | **p<0.001** | **p = 0.007** |
|  | **0.012 (0.014)** | **0.006 (0.007)** | **0.010 (0.013)** | -0.005 (0.082) | **0.018 (0.014)** | **0.014 (0.018)** |
| LBF | t(14) = 1.228 | **t(15) = 2.896** | **t(14) = 2.949** | **t(14) = 2.737** | **t(15) = 2.222** | **t(14) = 3.291** |
|  | p = 0.240 | **p = 0.011** | **p = 0.011** | **p = 0.016** | **p = 0.042** | **p = 0.005** |
|  | 0.005 (0.015) | **0.003 (0.004)** | **0.005 (0.006)** | **0.007 (0.010)** | **0.005 (0.010)** | **0.005 (0.006)** |
| RBF | t(14) = 2.067 | **t(15) = 2.142** | t(14) = 0.454 | **t(14) = 2.763** | **t(15) = 2.936** | **t(14) = 2.753** |
|  | p = 0.058 | **p = 0.049** | p =  0.595 | **p = 0.015** | **p = 0.010** | **p = 0.016** |
|  | <0.001 (0.001) | **0.001 (0.002)** | <0.001 (0.002) | **0.002 (0.003)** | **0.002 (0.002)** | **0.003 (0.005)** |
| LGAM | **t(14) = 3.052** | t(15) = 1.656 | **t(14) = 3.046** | **t(14) = 2.460** | **t(15) = 2.233** | t(14) = 1.363 |
|  | **p = 0.009** | p =  0.118 | **p = 0.009** | **p = 0.028** | **p = 0.041** | p = 0.194 |
|  | **0.013 (0.016)** | 0..012 (0.028) | **0.010 (0.012)** | **0.003 (0.005)** | **0.003 (0.005)** | 0..002 (0.005) |
| RGAM | t(14) = 1.623 | **t(15) = 3.612** | **t(14) = 2.626** | t(14) = 1.916 | **t(15) = 3.000** | **t(14) = 2.769** |
|  | p = 0.127 | **p = 0.003** | **p = 0.020** | p = 0.076 | **p = 0.009** | **p = 0.015** |
|  | 0.004 (0.009) | **0.004 (0.004)** | **0.006 (0.009)** | <0.001 (0.006) | **0.002 (0.003)** | **0.004 (0.005)** |
| LTA | t(14) = 1.591 | **t(15) = 2.455** | **t(14) = 2.460** | **t(14) = 3.273** | **t(15) = 2.6674** | **t(14) = 2.476** |
|  | p = 0.134 | **p = 0.027** | **p = 0.027** | **p = 0.006** | **p = 0.017** | **0.027** |
|  | 0.007 (0.009) | **0.006 (0.010)** | **0.002 (0.003)** | **0.019 (0.023)** | **0.012 (0.017)** | **0.010 (0.016)** |
| RTA | **t(14) = 3.054** | **t(14) = 2.605** | **t(14) = 2.172** | **t(14) = 2.689** | **t(15) = 3.935** | **t(14) = 3.191** |
|  | **p = 0.009** | **p = 0.021** | **p = 0.048** | **p = 0.018** | **p = 0.001** | **p = 0.007** |
|  | **0.007 (0.009)** | **0.016 (0.023)** | **0.005 (0.009)** | **0.015 (0.034)** | **0.011 (0.011)** | **0.011 (0.013)** |

Table S.3: Results of two-way RM-ANOVAs (factor1: wall angle; factor2: position). If sphericity condition is not met, Greenhouse-Geisser corrections are applied. Statistically significant results are highlighted in bold.

| **Variable** | **Wall angle** | **Position** | **Wall angle * Position** |
| --- | --- | --- | --- |
| HAND | **F(1.20,15.60) = 268.594** | **F(1,13) = 386.386** | **F(1.42,18.41) = 83.392** |
|  | **p < 0.001** | **p < 0.001** | **p < 0.001** |
| LF | **F(1.23,15.99) = 10.483** | **F(1,13) = 352.103** | **F(1.42,18.43) = 4.626** |
|  | **p = 0.004** | **p < 0.001** | **p = 0.034** |
| RF | **F(1.33,17.27) = 47.518** | **F(1,13) = 29.984** | F(1.22,15.86) = 4.160 |
|  | **p < 0.001** | **p < 0.001** | p = 0.052 |
| TF | **F(1.14,14.84) = 96.472** | **F(1,13) = 234.250** | **F(1.22,15.89) = 31.895** |
|  | **p < 0.001** | **p < 0.001** | **p < 0.001** |
| TRAP | **F(2,26) = 11.453** | F(1,13) = 1.444 | F(1,13) = 1.216 |
|  | **p < 0.001** | p = 0.251 | p = 0.313 |
| AD | **F(1.42,18.51) = 8.594** | **F(1,13) = 6.423** | **F(1.37,17.84) = 7.713** |
|  | **p = 0.005** | **p = 0.025** | **p = 0.008** |
| PD | **F(1.10,14.30) = 27.749** | F(1,13) = 0.321 | **F(2,26) = 12.251** |
|  | **p < 0.001** | p = 0.581 | **p < 0.001** |
| PM |  |  |  |
|  | F(1.03, 13.36) = 4.513  p = 0.052 | F(1,13) = 1.421  p = 0.255 | F(1.04,13.52) = 1.519  p = 0.240 |
| ISPIN | **F(2,26) = 21.499** | F(1,13) = 3.310 | **F(2,26) = 7.063** |
|  | **p < 0.001** | p = 0.092 | **p = 0.004** |
| LD | **F(1.32,17.10) = 55.513** | **F(1,13) = 14.092** | **F(2,26) = 34.490** |
|  | **p < 0.001** | **p = 0.002** | **p < 0.001** |
| ES | **F(2,26) = 57.109** | **F(1,13) = 34.806** | **F(1.07,13.94) = 12.859** |
|  | **p < 0.001** | **p < 0.001** | **p = 0.003** |
| BB | **F(1.09,14.19) = 36.494** | **F(1,13) = 15.130** | **F(1.15,14.94) = 16.529** |
|  | **p < 0.001** | **p = 0.002** | **p < 0.001** |
| TB | **F(1.09,14.17) = 35.688** | F(1,13) = 1.201 | F(1.10,14.26) = 3.088 |
|  | **p < 0.001** | p = 0.293 | p = 0.098 |
| BRAD | **F(1.26,16.33) = 89.781** | F(1,13) = 3.396 | **F(1.18,15.38) = 31.123** |
|  | **p < 0.001** | p = 0.088 | **p < 0.001** |
| FULN | **F(1.24,16.12) = 79.476** | **F(1,13) = 22.561** | **F(2,26) = 12.408** |
|  | **p < 0.001** | **p < 0.001** | **p < 0.001** |
| FRAD | **F(1.13,14.62) = 64.181** | **F(1,13) = 14.954** | **F(1.21,15.68) = 10.086** |
|  | **p < 0.001** | **p = 0.002** | **p = 0.004** |
| LGM | **F(1.19,15.43) = 49.674** | **F(1,13) = 5.884** | F(1.05,13.70) = 3.215 |
|  | **p < 0.001** | **p = 0.031** | p = 0.094 |
| RGM | **F(2,26) = 4.512** | **F(1,13) = 14.499** | F(1.32,17.14) = 1.360 |
|  | **p = 0.021** | **p = 0.002** | p = 0.271 |
| LRF | **F(1.33,17.27) = 13.258** | **F(1,13) = 8.260** | **F(2,26) = 6.171** |
|  | **p < 0.001** | **p = 0.013** | **p = 0.006** |
| RRF | **F(1.30,16.91) = 21.589** | F(1,13) = 1.541 | F(1.31,16.97) = 3.387 |
|  | **p < 0.001** | p = 0.236 | p = 0.074 |
| LBF | **F(1.23,16.01) = 43.001** | F(1,13) = 0.160 | **F(1.40,18.20) = 10.894** |
|  | **p < 0.001** | p = 0.696 | **p = 0.002** |
| RBF |  |  |  |
|  | F(2,26) = 2.681  p = 0.098 | F(1,13) = 1.406  p = 0.257 | F(1.27,16.49) = 0.084  p = 0.833 |
| LGAM | **F(1.21,15.68) = 9.755** | **F(1,13) = 24.709** | **F(1.41,18.28) = 5.442** |
|  | **p = 0.005** | **p < 0.001** | **p = 0.022** |
| RGAM | F(2,26) = 3.109 | **F(1,13) = 8.434** | F(2,26) = 1.308 |
|  | p = 0.062 | **p = 0.012** | p = 0.287 |
| LTA | **F(1.26,16.36) = 15.349** | **F(1,13) = 13.096** | F(1.26,16.41) = 3.869 |
|  | **p < 0.001** | **p = 0.003** | p = 0.058 |
| RTA | **F(1.18,15.33) = 13.326** | **F(1,13) = 7.462** | F(2,26) = 0.382 |
|  | **p = 0.001** | **p = 0.018** | p = 0.686 |

Table S.4: Results of the pairwise comparisons (Mean difference (std error)) computed using Bonferroni corrections as the wall angle changes. Statistically significant differences are highlighted in bold.

| **Variable** | **UP** | | | **DOWN** | | |
| --- | --- | --- | --- | --- | --- | --- |
|  | **Wall angle *i*** | **Wall angle *j*** | **Mean diff. *i-j*  (std err, p-value)** | **Wall angle *i*** | **Wall angle *j*** | **Mean diff. *i-j*  (std err, p-value)** |
| HAND | SLAB | VERTICAL | **-0.074 (0.007, <0.001)** | SLAB | VERTICAL | **-0.040 (0.004, <0.001)** |
|  | SLAB | OVERHANG | **-0.175 (0.011, <0.001)** | SLAB | OVERHANG | **-0.085 (0.005, <0.001)** |
|  | VERTICAL | OVERHANG | **-0.101 (0.006, <0.001)** | VERTICAL | OVERHANG | **-0.045 (0.004, <0.001)** |
| LF | SLAB | VERTICAL | -0.001 (0.008, 1.000) | SLAB | VERTICAL | 0.006 (0.004, 0.530) |
|  | SLAB | OVERHANG | 0.043 (0.016, 0.057) | SLAB | OVERHANG | **0.017 (0.006, 0.048)** |
|  | VERTICAL | OVERHANG | **0.045 (0.011, 0.005)** | VERTICAL | OVERHANG | **0.011 (0.004, 0.023)** |
| RF | SLAB | VERTICAL | **0.053 (0.008, <0.001)** | SLAB | VERTICAL | **0.026 (0.002, <0.001)** |
|  | SLAB | OVERHANG | **0.082 (0.016, <0.001)** | SLAB | OVERHANG | **0.049 (0.004, <0.001)** |
|  | VERTICAL | OVERHANG | 0.029 (0.012, 0.096) | VERTICAL | OVERHANG | **0.023 (0.004, <0.001)** |
| TF | SLAB | VERTICAL | **0.052 (0.006, <0.001)** | SLAB | VERTICAL | **0.032 (0.004, <0.001)** |
|  | SLAB | OVERHANG | **0.125 (0.013, <0.001)** | SLAB | OVERHANG | **0.066 (0.008, <0.001)** |
|  | VERTICAL | OVERHANG | **0.073 (0.007, <0.001)** | VERTICAL | OVERHANG | **0.034 (0.006, <0.001)** |
| TRAP | SLAB | VERTICAL | **-0.033 (0.024, 0.038)** | SLAB | VERTICAL | -0.030 (0.015, 0.192) |
|  | SLAB | OVERHANG | **-0.082 (0.019, 0.002)** | SLAB | OVERHANG | -0.046 (0.025, 0.266) |
|  | VERTICAL | OVERHANG | **-0.049 (0.012, 0.004)** | VERTICAL | OVERHANG | -0.016 (0.024, 1.000) |
| AD | SLAB | VERTICAL | 0.009 (0.006, 0.435) | SLAB | VERTICAL | **-0.028 (0.009, 0.024)** |
|  | SLAB | OVERHANG | -0.007 (0.008, 1.000) | SLAB | OVERHANG | **-0.053 (0.016, 0.015)** |
|  | VERTICAL | OVERHANG | **-0.017 (0.004, 0.006)** | VERTICAL | OVERHANG | -0.025 (0.011, 0.117) |
| PD | SLAB | VERTICAL | **-0.083 (0.015, <0.001)** | SLAB | VERTICAL | -0.052 (0.021, 0.076) |
|  | SLAB | OVERHANG | **-0.214 (0.030, <0.001)** | SLAB | OVERHANG | -0.080 (0.035, 0.117) |
|  | VERTICAL | OVERHANG | **-0.131 (0.021, <0.001)** | VERTICAL | OVERHANG | -0.028 (0.018, 0.437) |
| PM | SLAB | VERTICAL | -0.019 (0.011, 0.273) | SLAB | VERTICAL | -0.002 (0.006, 1.000) |
|  | SLAB | OVERHANG | -0.056 (0.028, 0.200) | SLAB | OVERHANG | -0.023 (0.016, 0.559) |
|  | VERTICAL | OVERHANG | -0.037 (0.018, 0.172) | VERTICAL | OVERHANG | -0.021 (0.012, 0.297) |
| ISPIN | SLAB | VERTICAL | **-0.167 (0.042, 0.005)** | SLAB | VERTICAL | **-0.042 (0.012, 0.016)** |
|  | SLAB | OVERHANG | **-0.208 (0.028, <0.001)** | SLAB | OVERHANG | **-0.103 (0.028, 0.008)** |
|  | VERTICAL | OVERHANG | -0.041 (0.029, 0.536) | VERTICAL | OVERHANG | -0.062 (0.034, 0.268) |
| LD | SLAB | VERTICAL | **-0.219 (0.040, <0.001)** | SLAB | VERTICAL | -0.062 (0.030, 0.187) |
|  | SLAB | OVERHANG | **-0.508 (0.061, <0.001)** | SLAB | OVERHANG | **-0.113 (0.032, 0.010)** |
|  | VERTICAL | OVERHANG | **-0.289 (0.035, <0.001)** | VERTICAL | OVERHANG | **-0.052 (0.014, 0.009)** |
| ES | SLAB | VERTICAL | **-0.087 (0.015, <0.001)** | SLAB | VERTICAL | **-0.035 (0.011, 0.021)** |
|  | SLAB | OVERHANG | **-0.181 (0.024, <0.001)** | SLAB | OVERHANG | **-0.081 (0.015, <0.001)** |
|  | VERTICAL | OVERHANG | **-0.094 (0.014, <0.001)** | VERTICAL | OVERHANG | **-0.046 (0.011, 0.003)** |
| BB | SLAB | VERTICAL | **-0.173 (0.039, 0.002)** | SLAB | VERTICAL | -0.019 (0.011, 0.359) |
|  | SLAB | OVERHANG | **-0.330 (0.059, <0.001)** | SLAB | OVERHANG | **-0.064 (0.021, 0.031)** |
|  | VERTICAL | OVERHANG | **-0.156 (0.025, <0.001)** | VERTICAL | OVERHANG | **-0.045 (0.012, 0.008)** |
| TB | SLAB | VERTICAL | **-0.017 (0.005, 0.028)** | SLAB | VERTICAL | **-0.011 (0.003, 0.011)** |
|  | SLAB | OVERHANG | **-0.043 (0.009, 0.001)** | SLAB | OVERHANG | **-0.024 (0.005, 0.002)** |
|  | VERTICAL | OVERHANG | **-0.026 (0.005, <0.001)** | VERTICAL | OVERHANG | **-0.013 (0.003, 0.004)** |
| BRAD | SLAB | VERTICAL | **-0.214 (0.030, <0.001)** | SLAB | VERTICAL | -0.075 (0.036, 0.183) |
|  | SLAB | OVERHANG | **-0.462 (0.032, <0.001)** | SLAB | OVERHANG | -0.088 (0.047, 0.248) |
|  | VERTICAL | OVERHANG | **-0.247 (0.019, <0.001)** | VERTICAL | OVERHANG | -0.013 (0.012, 0.880) |
| FULN | SLAB | VERTICAL | **-0.125 (0.018, <0.001)** | SLAB | VERTICAL | **-0.093 (0.019, 0.001)** |
|  | SLAB | OVERHANG | **-0.320 (0.039, <0.001)** | SLAB | OVERHANG | **-0.161 (0.026, <0.001)** |
|  | VERTICAL | OVERHANG | **-0.195 (0.027, <0.001)** | VERTICAL | OVERHANG | **-0.067 (0.019, 0.011)** |
| FRAD | SLAB | VERTICAL | **-0.157 (0.028, <0.001)** | SLAB | VERTICAL | **-0.093 (0.019, <0.001)** |
|  | SLAB | OVERHANG | **-0.385 (0.063, <0.001)** | SLAB | OVERHANG | **-0.145 (0.027, <0.001)** |
|  | VERTICAL | OVERHANG | **-0.228 (0.041, <0.001)** | VERTICAL | OVERHANG | **-0.052 (0.018, 0.037)** |
| LGM | SLAB | VERTICAL | **-0.069 (0.011, < 0.001)** | SLAB | VERTICAL | **-0.036 (0.011, 0.016)** |
|  | SLAB | OVERHANG | **-0.171 (0.026, < 0.001)** | SLAB | OVERHANG | **-0.101 (0.027, 0.007)** |
|  | VERTICAL | OVERHANG | **-0.102 (0.016, < 0.001)** | VERTICAL | OVERHANG | **-0.066 (0.020, 0.016)** |
| RGM | SLAB | VERTICAL | 0.001 (0.002, 1.000) | SLAB | VERTICAL | 0.004 (0.011, 1.000) |
|  | SLAB | OVERHANG | -0.006 (0.004, 0.617) | SLAB | OVERHANG | -0.023 (0.015, 0.468) |
|  | VERTICAL | OVERHANG | -0.007 (0.004, 0.300) | VERTICAL | OVERHANG | **-0.027 (0.010, 0.044)** |
| LRF | SLAB | VERTICAL | -0.003 (0.003, 1.000) | SLAB | VERTICAL | 0.001 (0.002, 1.000) |
|  | SLAB | OVERHANG | **-0.018 (0.005, 0.011)** | SLAB | OVERHANG | -0.001 (0.003, 1.000) |
|  | VERTICAL | OVERHANG | **-0.015 (0.004, 0.003)** | VERTICAL | OVERHANG | -0.001 (0.002, 1.000) |
| RRF | SLAB | VERTICAL | **-0.059 (0.012, 0.001)** | SLAB | VERTICAL | -0.004 (0.018, 1.000) |
|  | SLAB | OVERHANG | **-0.140 (0.021, < 0.001)** | SLAB | OVERHANG | -0.049 (0.037, 0.622) |
|  | VERTICAL | OVERHANG | **-0.081 (0.015, < 0.001)** | VERTICAL | OVERHANG | -0.044 (0.026, 0.324) |
| LBF | SLAB | VERTICAL | **-0.045 (0.010, 0.002)** | SLAB | VERTICAL | **-0.023 (0.006, 0.008)** |
|  | SLAB | OVERHANG | **-0.126 (0.019, < 0.001)** | SLAB | OVERHANG | **-0.057 (0.013, 0.003)** |
|  | VERTICAL | OVERHANG | **-0.081 (0.010, < 0.001)** | VERTICAL | OVERHANG | -0.034 (0.013, 0.061) |
| RBF | SLAB | VERTICAL | 0.004 (0.003, 0.497) | SLAB | VERTICAL | 0.002 (0.003, 1.000) |
|  | SLAB | OVERHANG | 0.007 (0.004, 0.274) | SLAB | OVERHANG | 0.006 (0.005, 0.660) |
|  | VERTICAL | OVERHANG | 0.003 (0.004, 1.000) | VERTICAL | OVERHANG | 0.004 (0.004, 0.814) |
| LGAM | SLAB | VERTICAL | -0.056 (0.023, 0.101) | SLAB | VERTICAL | -0.015 (0.006, 0.086) |
|  | SLAB | OVERHANG | **-0.117 (0.037, 0.021)** | SLAB | OVERHANG | -0.032 (0.015, 0.171) |
|  | VERTICAL | OVERHANG | **-0.061 (0.020, 0.025)** | VERTICAL | OVERHANG | -0.017 (0.012, 0.555) |
| RGAM | SLAB | VERTICAL | 0.027 (0.011, 0.086) | SLAB | VERTICAL | 0.004 (0.005, 1.000) |
|  | SLAB | OVERHANG | 0.014 (0.015, 1.000) | SLAB | OVERHANG | 0.001 (0.008, 1.000) |
|  | VERTICAL | OVERHANG | -0.013 (0.009, 0.538) | VERTICAL | OVERHANG | -0.003 (0.006, 1.000) |
| LTA | SLAB | VERTICAL | -0.015 (0.009, 0.364) | SLAB | VERTICAL | -0.047 (0.017, 0.057) |
|  | SLAB | OVERHANG | -0.040 (0.018, 0.147) | SLAB | OVERHANG | **-0.102 (0.025, 0.004)** |
|  | VERTICAL | OVERHANG | -0.024 (0.012, 0.190) | VERTICAL | OVERHANG | **-0.056 (0.016, 0.011)** |
| RTA | SLAB | VERTICAL | -0.029 (0.014, 0.199) | SLAB | VERTICAL | -0.010 (0.010, 1.000) |
|  | SLAB | OVERHANG | -0.064 (0.031, 0.181) | SLAB | OVERHANG | -0.037 (0.018, 0.188) |
|  | VERTICAL | OVERHANG | -0.035 (0.018, 0.233) | VERTICAL | OVERHANG | -0.027 (0.012, 0.107) |

Table S.5: Results of the pairwise comparisons (Mean difference (std error)) computed using Bonferroni corrections as the position changes. Statistically significant differences are highlighted in bold.

| **Variable** | **Wall angle** | **Mean diff. UP-DOWN (std error, p-value)** |
| --- | --- | --- |
| HAND | SLAB | **-0.321 (0.017, <0.001)** |
|  | VERTICAL | **-0.287 (0.013, <0.001)** |
|  | OVERHANG | **-0.230 (0.014, <0.001)** |
| LF | SLAB | **0.204 (0.012, <0.001)** |
|  | VERTICAL | **0.211 (0.013, <0.001)** |
|  | OVERHANG | **0.178 (0.012, <0.001)** |
| RF | SLAB | **0.093 (0.015, <0.001)** |
|  | VERTICAL | **0.065 (0.012, <0.001)** |
|  | OVERHANG | **0.060 (0.017, 0.004)** |
| TF | SLAB | **0.297 (0.019, <0.001)** |
|  | VERTICAL | **0.276 (0.018, <0.001)** |
|  | OVERHANG | **0.238 (0.017, <0.001)** |
| TRAP | SLAB | 0.009 (0.019, 0.648) |
|  | VERTICAL | 0.013 (0.024, 0.608) |
|  | OVERHANG | 0.045 (0.027, 0.117) |
| AD | SLAB | **-0.107 (0.048, 0.043)** |
|  | VERTICAL | **-0.144 (0.052, 0.016)** |
|  | OVERHANG | **-0.153 (0.060, 0.024)** |
| PD | SLAB | -0.034 (0.024, 0.172) |
|  | VERTICAL | -0.003 (0.043, 0.948) |
|  | OVERHANG | 0.100 (0.050, 0.067) |
| PM | SLAB | -0.007 (0.011, 0.521) |
|  | VERTICAL | 0.010 (0.007, 0.174) |
|  | OVERHANG | 0.026 (0.019, 0.208) |
| ISPIN | SLAB | 0.007 (0.049, 0.882) |
|  | VERTICAL | **0.133 (0.050, 0.019)** |
|  | OVERHANG | 0.112 (0.053, 0.055) |
| LD | SLAB | -0.034 (0.034, 0.348) |
|  | VERTICAL | **0.124 (0.052, 0.033)** |
|  | OVERHANG | **0.361 (0.057, <0.001)** |
| ES | SLAB | **-0.184 (0.028, <0.001)** |
|  | VERTICAL | **-0.131 (0.023, <0.001)** |
|  | OVERHANG | **-0.084 (0.025, 0.005)** |
| BB | SLAB | 0.012 (0.009, 0.170) |
|  | VERTICAL | **0.167 (0.046, 0.003)** |
|  | OVERHANG | **0.279 (0.068, 0.001)** |
| TB | SLAB | **-0.016 (0.007, 0.031)** |
|  | VERTICAL | -0.011 (0.009, 0.228) |
|  | OVERHANG | 0.002 (0.011, 0.858) |
| BRAD | SLAB | **-0.073 (0.031, 0.036)** |
|  | VERTICAL | 0.067 (0.065, 0.322) |
|  | OVERHANG | **-0.301 (0.075, 0.002)** |
| FULN | SLAB | **-0.373 (0.061, <0.001)** |
|  | VERTICAL | **-0.341 (0.066, <0.001)** |
|  | OVERHANG | **-0.213 (0.075, 0.014)** |
| FRAD | SLAB | **-0.337 (0.053, <0.001)** |
|  | VERTICAL | **-0.273 (0.066, 0.001)** |
|  | OVERHANG | -0.097 (0.084, 0.264) |
| LGM | SLAB | **-0.116 (0.024, < 0.001)** |
|  | VERTICAL | **-0.083 (0.031, 0.018)** |
|  | OVERHANG | -0.046 (0.052, 0.384) |
| RGM | SLAB | **-0.099 (0.024, 0.001)** |
|  | VERTICAL | **-0.096 (0.026, 0.003)** |
|  | OVERHANG | **-0.116 (0.034, 0.005)** |
| LRF | SLAB | 0.008 (0.006, 0.207) |
|  | VERTICAL | 0.011 (0.005, 0.064) |
|  | OVERHANG | **0.025 (0.006, 0.002)** |
| RRF | SLAB | **-0.081 (0.028, 0.012)** |
|  | VERTICAL | -0.026 (0.025, 0.326) |
|  | OVERHANG | 0.011 (0.043, 0.804) |
| LBF | SLAB | -0.040 (0.028, 0.181) |
|  | VERTICAL | -0.019 (0.025, 0.475) |
|  | OVERHANG | 0.029 (0.025, 0.267) |
| RBF | SLAB | -0.014 (0.014, 0.338) |
|  | VERTICAL | -0.016 (0.014, 0.295) |
|  | OVERHANG | -0.015 (0.009, 0.139) |
| LGAM | SLAB | **0.104 (0.031, 0.005)** |
|  | VERTICAL | **0.145 (0.035, 0.001)** |
|  | OVERHANG | **0.189 (0.032, < 0.001)** |
| RGAM | SLAB | **0.055 (0.020, 0.019)** |
|  | VERTICAL | **0.031 (0.013, 0.032)** |
|  | OVERHANG | **0.040 (0.016, 0.026)** |
| LTA | SLAB | **-0.031 (0.014, 0.045)** |
|  | VERTICAL | **-0.062 (0.021, 0.013)** |
|  | OVERHANG | **-0.094 (0.027, 0.004)** |
| RTA | SLAB | -0.063 (0.030, 0.053) |
|  | VERTICAL | **-0.044 (0.020, 0.042)** |
|  | OVERHANG | -0.037 (0.026, 0,174) |
